# Parallel disease activity of Behçet’s disease with renal and entero involvements: a case report

**DOI:** 10.1186/s12882-021-02327-9

**Published:** 2021-04-07

**Authors:** Kanako Watanabe-Kusunoki, Masaru Kato, Yotaro Oki, Tetsuo Shimizu, Yoshihiro Kusunoki, Shota Furukawa, Shin Furukawa, Hirohiko Kitakawa, Kiyoshi Sakai

**Affiliations:** 1 Department of Internal Medicine, Kushiro Red Cross Hospital, 21-14, Shinei-cho, Kushiro, 085-8512 Japan; 2 Department of Rheumatology, Nephrology and Endocrinology, Faculty of Medicine and Graduate School of Medicine, Hokkaido University, Sapporo, Japan

**Keywords:** Behçet’s disease, Renal involvement, Minimal change disease, Spontaneous remission, Parallel disease activity

## Abstract

**Background:**

Behçet’s disease (BD) is a systemic inflammatory vasculitis with both autoimmune and autoinflammatory properties. Renal involvement in BD and its spontaneous remission have been rare. We herein describe a case of parallel disease activity of BD with entero and renal involvements, followed by a spontaneous remission without corticosteroid treatment.

**Case presentation:**

A 54-year-old woman who had a 4-year history of BD, maintained with colchicine treatment, suffered abdominal pain, hemorrhagic stool and diarrhea. Physical examination revealed strong tenderness in the entire abdomen. Laboratory test results showed increased levels of inflammation, and a computed tomography scan revealed edematous intestinal wall thickening with ascites. Blood and stool cultures showed no specific findings. Since she was suspected to have developed panperitonitis with acute enterocolitis, she started treatment with an antibacterial agent under bowel rest. Her abdominal symptoms gradually improved, while diarrhea and high levels of inflammatory reaction persisted. Colonoscopy revealed discontinuous abnormal mucosal vascular patterns and ulcerations in the whole colon except for the rectum, and histological analyses of the intestine demonstrated transmural mucosal infiltration of inflammatory cells without epithelioid granuloma or amyloid deposition. Based on these findings, she was diagnosed with entero BD. Meanwhile, pedal edema appeared during her hospitalization. Urinalysis results were consistent with nephrotic syndrome, thus a renal biopsy was performed. Light microscopy showed no obvious glomerular and interstitial abnormalities, whereas electron microscopy revealed foot process effacement without immune complex deposition or fibrillary structure, compatible with minimal change disease (MCD). Only with conservative therapy, her proteinuria decreased, followed by a complete remission in 3 weeks from the onset of edema. The coincident episode of MCD was finally diagnosed as renal BD that paralleled disease activity to entero BD. She started adalimumab administration, resulting in the further improvement of diarrhea and inflammatory levels.

**Conclusions:**

This is the first report to demonstrate MCD as renal involvement of BD along with the disease activity of entero BD.

## Background

Behçet’s disease (BD) is a systemic inflammatory vasculitis characterized by oral aphthous ulcers, genital ulcers, nodular skin lesions, ocular lesions, and other atypical manifestations such as gastrointestinal, neurological and cardiovascular abnormalities [1]. Although the pathogenesis of BD is unclear, the activation of both innate and adaptive immunity plays an important role in the development of BD [2]. BD has been classified as the intersection of autoimmune and autoinflammatory syndromes that show unprovoked exacerbation and remission of inflammatory episodes. Although renal involvement in BD is relatively rare, there has been an increasing number of reports showing a connection [3]. Among the renal BD, glomerular disease is relatively rare, especially as there have been only a few reports that describe a case of minimal change disease (MCD). Here we present a case of parallel disease activity of entero and renal BD, diagnosed as MCD, followed by a spontaneous complete remission without corticosteroid treatment.

## Case presentation

A 54-year-old Japanese woman who had a 4-year history of BD suffered abdominal pain, hemorrhagic stool and diarrhea. BD was diagnosed based on the presence of oral and genital ulcers and erythema nodosum, and carriage of human leukocyte antigens (HLA)-B51. She started taking prednisolone at 20 mg/day and colchicine, resulting in disease remission. Prednisolone was tapered down and discontinued in a year, while colchicine had been continued for maintenance therapy. She was admitted to our department for examination and treatment for abdominal symptoms.

**Fig. 1:**
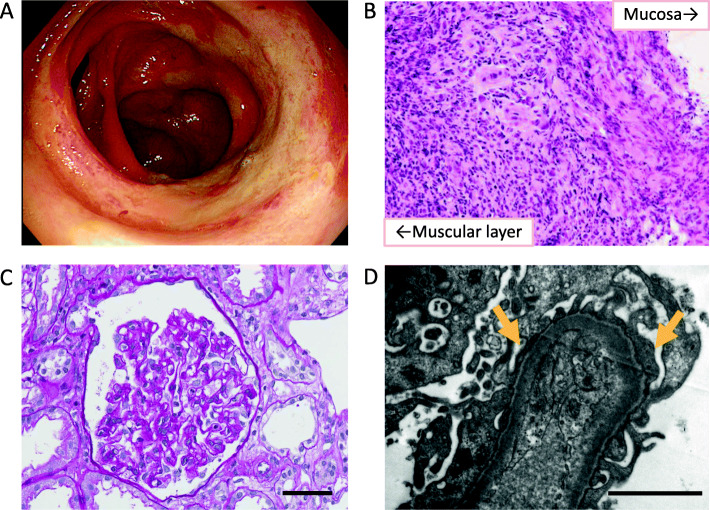
Findings of colonoscopy, intestinal biopsy and renal biopsy. **a** Discontinuous abnormal mucosal vascular patterns and ulcerations were detected in the cecum, ascending colon, transverse colon, descending colon and sigmoid colon by colonoscopy. **b** Histological analyses of the intestine showed transmural mucosal infiltration of inflammatory cells including lymphocytes and neutrophils. **c** The Periodic acid-Schiff staining of the kidney showed no glomerular and interstitial abnormalities. Scale bar, 50 μm. **d** Electron microscopy of the kidney revealed foot process effacement (arrowheads) without immune complex deposition or fibrillary structure. Scale bar, 2 μm

Physical examination revealed strong tenderness in the entire abdomen. Laboratory test results (Table 1) showed elevated levels of white blood cell counts (WBC; 32,080 /μL) and C-reactive protein (CRP; 26.7 mg/dL), while decreased levels of serum albumin (2.9 g/dL). The interferon-gamma release assay was negative. A computed tomography scan revealed edematous intestinal wall thickening with ascites. Blood cultures from separate sampling showed no microbial growth, and stool culture did not result in the growth of any specific bacteria that cause enteritis such as enteropathogenic *Escherichia coli* or *Campylobacter*. Based on the findings of physical examination, laboratory test results and imaging studies, she was suspected to have developed panperitonitis with acute enterocolitis. Therefore, we started her treatment with an antibacterial agent (Meropenem) under bowel rest, and required analgesics (Acetaminophen) for 1 week. Her abdominal pain and hemorrhagic stool gradually improved, however, diarrhea and high levels of CRP persisted. The antibacterial agent was discontinued on day 18. Colonoscopy on day 24 revealed discontinuous abnormal mucosal vascular patterns and ulcerations in the cecum, ascending colon, transverse colon, descending colon and sigmoid colon (Fig. 1a). Histological analyses of the intestine showed transmural mucosal infiltration of inflammatory cells including lymphocytes and neutrophils, without the findings of epithelioid granuloma or amyloid deposition (Fig. 1b). Based on these findings, the patient was diagnosed with entero BD.

**Table 1:** Laboratory data

**Complete Blood Count**		
WBC	32,080	/μL
RBC	5.56	× 10^6^/μL
Hemoglobin	10.8	g/dL
Platelet	35.4	× 10^4^/μL
**Biochemistry**		
TP	6.4	g/dL
Alb	2.9	g/dL
LDH	253	U/L
BUN	41.9	mg/dL
Cr	2.09	mg/dL
eGFR	20.4	mL/min/1.73m^2^
CRP	26.7	mg/dL
**Infection**		
HBs Ag	(−)	
HBV Ab	(−)	
IGRA	(−)	
**Immunoserological test (10 days after admission)**		
IgG	949	mg/dL
IgA	244	mg/dL
IgM	119	mg/dL
C3	106	mg/dL
C4	25	mg/dL
CH50	64	U/mL
ANA	< 40	times
ASO	< 20	IU/mL
ASK	< 20	times
PR3-ANCA	< 1	U/mL
MPO-ANCA	< 1	U/mL
**Urinalysis (10 days after admission)**		
Hematuria	1–4	/HPF
Proteinuria	7.42	g/gCr
Bence-Jones protein	(−)	

*ANA* Antinuclear antibody, *ANCA* Antineutrophil cytoplasmic antibody, *ASO* Anti-streptolysin O antibody, *ASK* Anti-streptokinase antibody, *IGRA* Interferon-gamma release assay

Meanwhile, pedal edema had appeared and had been exacerbated from day 7. Urinalyses showed high levels of proteinuria (7.42 urine protein to urine creatinine ratio) that was consistent with nephrotic syndrome (Table 1). Given the concurrent development of proteinuria and entero-BD and no abnormal findings from the immunoserological test (Table 1), we suspected BD-associated glomerulonephritis and thus performed a renal biopsy. Light microscopy showed no obvious glomerular or interstitial abnormalities, whereas electron microscopy revealed foot process effacement without immune complex deposition or fibrillary structure (Fig. 1c and d), compatible with MCD. Only with conservative therapy, her proteinuria turned to decrease and her pedal edema gradually improved. After 3 weeks from the onset of pedal edema, the patient had achieved complete remission without any additional treatment.

**Fig. 2:**
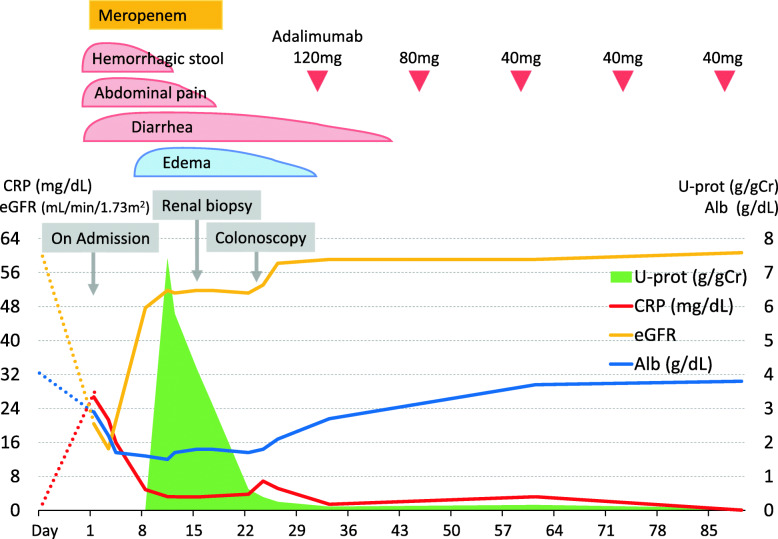
Clinical course. Alb, albumin; CRP, C-reactive protein; U-prot, urine protein

The coincident episode of MCD was finally diagnosed as renal BD that paralleled disease activity to entero BD. In order to manage the activity of BD and maintain the remission of renal BD, she was administered adalimumab (initial doses of 120 mg and 80 mg on days 32 and 46, respectively, followed by 40 mg every other week), resulting in the further improvement of diarrhea and the serum levels of CRP. Both entero and renal BD maintained in remission during a 3-month follow-up (Fig. 2).

## Discussion and conclusions

The present case of MCD with a rare manifestation of renal involvement of BD showed spontaneous complete remission in 3 weeks from the onset of pedal edema without corticosteroid treatment, paralleled with the disease activity of entero BD. This report indicates the potential of renal BD to show remission without specific treatment depending on the activity of BD.

Although the pathogenesis of BD is obscure, genetic factors and immunological aberrations have been shown to play an important role in the development and progression of BD [2]. Various immune cells and cytokines released by activated innate and adaptive immune systems, including autoimmune regulatory T (Treg) cells and type 22 T helper (Th22) cells, have been reported to be involved in the immunopathogenesis of BD. The Th17/Treg balance is important in the regulation of inflammation in patients with active BD [4], while increased Th22-type cytokines and cells have shown to be involved in the acute immune response in BD [5, 6]. With respect to MCD, circulating mediators produced by abnormal T cells are thought to be related to its development. The overexpression of interleukin-13 (IL-13), which is produced mainly by Th2 cells and partly by Th22 cells [7], induced MCD-like disease with foot process effacement and proteinuria [8]. Moreover, a hypofunction of Treg cells has been shown to be crucial for the development of MCD [9]. Based on these reports, we hypothesize that a malfunction of Treg cells or increased levels of IL-13 produced in part by Th22 cells in BD might be related to the development of MCD as the common etiology.

The frequency of renal manifestations in BD is reported to vary from less than 1 to 29% with wide clinical and histological spectrums. The underlying pathological changes in the kidney are classified into five groups; (a) amyloidosis, (b) glomerulonephritis, (c) renal vascular involvement, (d) interstitial nephritis, and (e) others such as drug-induced nephrotoxicity. Treatment of renal BD depends on the pathological changes and other organ involvements; corticosteroids, colchicine, azathioprine and cyclophosphamide have been used in the management of glomerulonephritis in BD. The prognosis of patients with glomerulonephritis in renal BD is favorable, with only a few cases known to have developed into end-stage renal diseases [3]. MCD is a rare manifestation in renal BD and only a few cases have been reported in literature to date [10, 11]. They developed in an active state of BD along with oral and genital ulcers and venous thrombosis, followed by the improvement using corticosteroid treatments.

In the present case, the patient was diagnosed with entero BD based on the findings of colonoscopy and histological analysis of intestinal biopsy; discontinuous ulcerations were observed throughout the colon including cecum with transmural mucosal infiltration of inflammatory cells, compatible with previous cases [12]. Infectious enteritis and other inflammatory bowel disease were excluded based on the clinical course and the findings of colonoscopy, histological analysis and cultivation tests. A coincident episode of MCD was diagnosed as renal BD that paralleled disease activity to intestinal involvement. No other secondary causes of MCD, such as infections, allergy, malignancies and drugs were detected [13].

Of note, our case with MCD in renal BD showed spontaneous remission in 3 weeks from the onset of symptoms without immunosuppressive therapy. There are a few cases of renal BD that showed spontaneous remission [14, 15]. These reports showed immunoglobulin A (IgA) nephropathy as a manifestation of renal BD that occurred during the inactive state of BD, followed by complete remission in 1 year from the diagnosis. Although it may be difficult to rule out primary IgA nephropathy, the reports indicate a possibility of renal BD to show spontaneous remission. A randomized trial that has explored the use of corticosteroids in MCD showed that about 60% of the patients with MCD in the control group experienced a spontaneous remission in 2 years, while there is a little decrease of proteinuria during the first month among control patients compared to patients with a corticosteroid-treatment group [16]. On the other hand, a patient with MCD associated with influenza B infection showed spontaneous remission within 2 weeks after the onset of symptoms only with conservative treatment [17]. Though more evidential reports should be accumulated, it is worth keeping in mind that the resolution of the cause of MCD might lead to early remission as with our case.

Tumor necrosis factor-α (TNF-α) is a representative pro-inflammatory cytokine produced by a wide range of immune cells and plays an important role in the induction and maintenance of inflammation in the autoimmune response. TNF-α antagonists, such as infliximab, adalimumab and etanercept, have shown to be an effective treatment for BD [1]. We so far discussed the present case of renal biopsy-proven MCD as secondary to BD because a) the disease peak matched to entero BD, b) no other secondary cause of MCD was detected, and c) the disease induced early remission without corticosteroid therapy. Since BD and MCD share the common pathogenesis of immunological aberrations, adalimumab would be a useful treatment for secondary MCD to BD in our case to maintain remission and manage the activity of BD itself, as etanercept showed effectiveness in a case of nephrotic syndrome due to focal segmental glomerulosclerosis in renal BD [18],

This is the first case report to demonstrate that MCD as renal involvement of BD showed spontaneous remission along with the disease activities of BD. Renal BD, having both properties of autoimmune diseases as well as autoinflammatory syndromes, may have the potential to show unprovoked remission without specific treatment depending on its states of disease.

## Data Availability

The datasets used and/or analysed during the current study are available from the corresponding author on reasonable request.

## Abbreviations

BD: Behçet’s disease
CRP: C-reactive protein
HLA: Human leukocyte antigen
IgA: Immunoglobulin A
IL-13: Interleukin-13
MCD: Minimal change disease
Th22: Type 22 T helper cells
TNF-α: Tumor necrosis factor-α
Treg: Regulatory T cells
WBC: White blood cell
